# Climatic suitability and distribution prediction of *Medicago sativa* on the Qinghai–Tibet Plateau: an ensemble species distribution modeling approach

**DOI:** 10.3389/fpls.2026.1930959

**Published:** 2026-09-14

**Authors:** Jing Xiong, Jianglin Kang, Yi Huang

**Affiliations:** 1 College of Artificial Intelligence, Mianyang Teachers’ College, Mianyang, China; 2 School of Geographical Sciences, China West Normal University, Nanchong, China

**Keywords:** climatic suitability, ensemble species distribution model, *Medicago sativa* L., potential distribution, Qinghai-Tibet Plateau

## Abstract

**Introduction:**

*Medicago sativa* L. is a widely cultivated forage species with potential applications in the phytoremediation of heavy metal-contaminated soils. Its application on the climate-sensitive Qinghai–Tibet Plateau depends on the distribution and future dynamics of climatically suitable habitats. This study aimed to identify the main environmental constraints on its potential distribution and assess changes in suitable habitats under future climate change.

**Methods:**

Using 92 spatially filtered occurrence records and 18 environmental variables, we developed species distribution models with 12 algorithms and constructed a weighted ensemble from models meeting the screening criterion. Potentially suitable habitats were projected for the current period and for three emission scenarios in the 2050s and 2090s, and key environmental variables and centroid shifts were analyzed.

**Results:**

Elevation and annual temperature range were the main environmental constraints. Under current conditions, moderate- and high-suitability habitats were concentrated mainly in the eastern and southern Qinghai–Tibet Plateau. Total suitable area generally increased under future scenarios, mainly through expansion of low- and moderate-suitability habitats, whereas high-suitability habitat increased more slowly and accounted for a smaller proportion of the total suitable area. Centroid shifts varied among periods and scenarios.

**Discussion:**

Climate change may reorganize the potential suitability pattern of *M. sativa* on the Qinghai–Tibet Plateau and provide a spatial basis for identifying candidate areas for ecological restoration. These projections represent climatic suitability rather than actual occurrence or phytoremediation effectiveness; practical application should therefore be validated against soil contamination, land use, and field conditions.

## 1 Introduction

The Qinghai–Tibet Plateau, often referred to as the Earth’s “Third Pole”, functions as a critical ecological barrier in China (Miao et al., 2024; Li et al., 2023). However, increasing resource exploitation, industrial development, and population growth in surrounding regions have intensified heavy-metal contamination in Plateau soils (Sheng et al., 2012; Wu et al., 2016). Since the 1970s, anthropogenic activities have contributed to substantial increases in heavy-metal concentrations in lake sediments and roadside soils, particularly in the northeastern Qinghai–Tibet Plateau (Wang et al., 2010; Yang et al., 2020). Among available remediation approaches, phytoremediation has received increasing attention because of its relatively low cost, limited secondary pollution, and potential to improve soil structure and ecological functions simultaneously (Xu et al., 2022; Hou et al., 2020; Xu et al., 2020). Its effectiveness, however, strongly depends on plant species selection, as species differ markedly in their tolerance to, uptake of, and stabilization capacity for heavy metals. Identifying plant species that are both environmentally adaptable and functionally suitable is therefore essential for the ecological restoration of heavy-metal-contaminated soils on the Qinghai–Tibet Plateau.

Numerous hyperaccumulator species can sequester large amounts of cadmium (Cd) in aboveground tissues and exhibit high remediation efficiency (Xu et al., 2020). However, their large-scale application is often constrained by low biomass, slow growth, and narrow ecological adaptability (Reeves et al., 2018). In contrast, *Medicago sativa* L. (*M. sativa*), one of the world’s most widely distributed forage crops (Zhou, 2013) (**Supplementary Figure 1**), has high biomass, rapid growth, broad adaptability, and the capacity to accumulate multiple heavy metals, including Cd, Cu, Pb, and Zn (Li et al., 2018; Zhou et al., 2025). As a perennial species adapted mainly to temperate and subtropical climates, *M. sativa* tolerates drought through its deep root system and grows well in well-drained, neutral to slightly alkaline soils, although its tolerance to extreme alpine cold is limited (Liu, 2025). Its deep rooting, nitrogen-fixing capacity, and stress tolerance further support its use in ecological restoration (Jiang et al., 2021). Compared with typical hyperaccumulators, *M. sativa* also shows strong Cd uptake potential, highlighting its value for phytoremediation of Cd-contaminated soils. Previous studies have explored the ecological adaptability of *M. sativa* under specific environmental conditions. For example, Chen et al. (2023) examined its effects on fungal communities in arid regions, while Lei (2021) evaluated its cultivation at mid-to-high elevations, suggesting a broad elevational tolerance. However, regional-scale responses of *M. sativa* to environmental gradients and climate change remain poorly understood, particularly on the ecologically sensitive Qinghai–Tibet Plateau. However, its application on the Qinghai–Tibet Plateau depends on the availability of suitable habitats under extreme cold, strong climatic seasonality, and steep elevational gradients. Assessing climatic suitability is therefore essential for evaluating its potential for large-scale phytoremediation.

Climate change is a major driver of species distribution shifts worldwide (Mi et al., 2023; Thomas et al., 2015; Brooks et al., 2006; Chen et al., 2015) as changes in temperature and precipitation alter plant phenology, productivity, and geographic ranges (Harvey et al., 2023; Schlaepfer and Lawler, 2023). Although plant distributions are jointly shaped by climate, topography, hydrology, and soil properties (Liu et al., 2022, Liu et al., 2021; Alkemade et al., 2011), climatic variables are major determinants of long-term biogeographic patterns and habitat suitability (Avasthi, 2005; Wan et al., 2024). Tibet Plateau, one of the world’s most climate-sensitive regions, has undergone substantial warming in recent decades (Zhang et al., 2024), potentially reshaping the suitable range of *M. sativa* and its applicability to ecological restoration. Assessing its climatic suitability under current and future conditions is therefore essential for evaluating its potential use in phytoremediation of Cd-contaminated soils.

Species Distribution Models (SDMs) are widely used to estimate potential habitats and assess distributional responses to climate change (Zhao et al., 2023; Xian et al., 2023; Huang et al., 2023; Shao et al., 2024; Huang et al., 2022). Because individual algorithms differ in predictive performance and sensitivity to environmental variables (Huang et al., 2023; Shao et al., 2024; Huang et al., 2022; Wen et al., 2022), ensemble approaches can reduce model-specific uncertainty. The Biomod2 platform integrates 12 commonly used algorithms to improve prediction robustness and stability (Thuiller et al., 2009; Hao et al., 2019), while enabling identification of key environmental drivers and projection of suitable habitats under multiple climate scenarios.

Although previous studies have examined the physiological responses and heavy-metal accumulation of *M. sativa*, its large-scale climatic suitability across the highly heterogeneous Qinghai–Tibet Plateau remains poorly understood. Therefore, this study aims to: (1) project the current and future potential distribution of *M. sativa* using an ensemble modeling approach; (2) identify the key environmental drivers shaping its distribution; and (3) assess climate-induced changes in habitat patterns and dynamics. Addressing these questions will help fill the knowledge gap in the climatic suitability of *M. sativa* in alpine regions. This study assesses climatic suitability rather than actual distribution, the former being a prerequisite for evaluating the species’ potential application in the remediation of heavy-metal-contaminated soils on the Qinghai–Tibet Plateau. Climatic suitability indicates potential habitat availability but does not guarantee successful establishment or remediation performance, which may also depend on land use, grazing pressure, soil contamination levels, and management practices and therefore require further field validation.

## 2 Materials and methods

### 2.1 Species distribution records

This study collected a total of 135 distribution records of *M. sativa* (data sources: National Plant Digital Specimen Resources (http://www.cvh.ac.cn/, accessed 1 May 2025), China Plant Encyclopedia (https://www.iplant.cn, accessed 1 May 2025), and China National Knowledge Infrastructure (https://www.cnki.net/, accessed 1 May 2025) (**Supplementary Table 2**). To reduce spatial autocorrelation, 5 km × 5 km grids were generated using the “Create Fishnet” tool in ArcGIS 10.4.1, and one record was randomly retained per grid using “Spatial Join” (Liu et al., 2022, Liu et al., 2021; Huang et al., 2022). After spatial filtering, 92 valid records remained (**Supplementary Figure 2**). Because *M. sativa* is widely cultivated, records from specimen and encyclopedia databases may partly reflect cultivation rather than natural climatic tolerance, introducing potential anthropogenic bias into the occurrence dataset.

### 2.2 Acquisition and processing of environmental variables

A total of 28 environmental variables were initially compiled, including 19 bioclimatic, five edaphic, three topographic, and one UV-B radiation variable (**Supplementary Table 1**). Current and future climate data were obtained from the WorldClim database (http://worldclim.org/data/index.html, accessed 1 May 2025). Future projections were based on the Beijing Climate Center Climate System Model (BCC-CSM2-MR) under three Shared Socioeconomic Pathway scenarios, SSP1-2.6, SSP3-7.0, and SSP5-8.5, representing low, intermediate, and high greenhouse-gas emission trajectories, respectively. BCC-CSM2-MR was selected because it participated in the sixth Coupled Model Intercomparison Project (CMIP6), has demonstrated strong simulation capability over China, and performs well in reproducing temperature and precipitation changes across the Qinghai–Tibet Plateau (Alem et al., 2026; Chen et al., 2024; Huang et al., 2026). Soil and topographic data were obtained from the FAO Harmonized World Soil Database (HWSD; http://www.fao.org/faostat/en/#data, accessed on 1 May 2025), and UV-B radiation data from the global UV-B radiation dataset (gIUV; https://www.ufz.de/gluv/index.php, accessed on 1 May 2025). All raster layers were resampled to 2.5 arc-min spatial resolution to ensure consistency among datasets.

To minimize multicollinearity among predictors, correlation and contribution analyses were conducted before model fitting (Song et al., 2023; Liu et al., 2024). First, all variables were entered into MaxEnt and evaluated over ten runs, and variables with 0% contribution were excluded. Pearson’s correlation analysis was then performed on the remaining predictors. For pairs with |*r*| ≥ 0.8, the variable with the higher contribution was retained (Yang et al., 2024; Allouche et al., 2006). After screening, 18 variables were retained for modeling, including nine climatic, five edaphic, three topographic, and one UV-B variable (**Supplementary Table 1**).

### 2.3 Model construction

Species distribution modeling was conducted using the Biomod2 package, which integrates 12 algorithms to construct ensemble models based on occurrence and pseudo-absence data. Biomod2 was selected because it supports weighted-average ensemble modeling to reduce single-algorithm bias (Thuiller et al., 2009), provides multiple evaluation metrics (TSS, ROC, and Kappa) and calibration procedures for cross-model comparison, and generally improves prediction stability and reliability relative to single-algorithm approaches such as MaxEnt (Hao et al., 2019). A total of 1,200 pseudo-absence points were randomly generated from the background environment using the “random” function. Following previous studies (Huang et al., 2025, Huang et al., 2026), a presence-to-pseudo-absence ratio of approximately 1:10–1:20 was adopted to improve model stability and reduce overfitting when occurrence records are limited.

In this study, hyperparameters were tuned using the BIOMOD_ModelingOptions function in the biomod2 package within predefined algorithm-specific ranges. For RF, ntree (500, 1000) and mtry (1-3) were tuned; for GBM, n.trees (1000-5000), shrinkage (0.01, 0.005), and interaction.depth (3-7) were tested; and for GLM, stepwise regression with and without quadratic terms was evaluated. Other algorithms used the default biomod2 tuning ranges. The optimal parameter set for each algorithm was selected based on the highest TSS from five-fold cross-validation. Models were calibrated using a 75/25 training-testing split, with equal weights assigned to presence and pseudo-absence data. Each model was replicated ten times, yielding 120 simulations in total.

Model performance was evaluated using the area under the receiver operating characteristic curve (ROC), Kappa, and True Skill Statistic (TSS). Filtering was applied at the replicate level; thus, some replicates of the same algorithm could be retained while others were excluded. Of the 120 simulations (12 algorithms × 10 replicates), only replicates with TSS ≥ 0.7 were included in the ensemble. Algorithms with consistently poor performance were excluded entirely, and the retained replicates were combined using a TSS-weighted average (Huang et al., 2026). The final ensemble prediction was generated by combining the results of individual models using a weighted average. In this study, the threshold was determined using the maximum training sensitivity plus specificity (MaxSS) method, a commonly used approach for binarizing SDM outputs into suitable/unsuitable areas (Huang et al., 2025). The MaxSS threshold of this ensemble model was 0.30; areas above this threshold were further divided at equal intervals into low (0.30–0.45), medium (0.45–0.65), and high (0.65–1.00) suitability classes to facilitate spatial comparisons among climate scenarios. The spatial outputs were visualized using ArcGIS 10.4.1.

Suitable habitat area was calculated from the ensemble-model suitability classes (high, medium, low, and unsuitable). The number of raster cells in each class was counted using the “Zonal Geometry” tool in ArcGIS 10.4.1 and multiplied by the area of a single cell (approximately 25 km²) to estimate the corresponding habitat area. Habitat centroids under different periods and climate scenarios were calculated using the SDMTool package (v1.1-21) in R. Centroid coordinates and migration distances were then determined in ArcGIS 10.4.1 to visualize migration paths and spatial trends. Base maps were obtained from the 1:1,000,000 public-version fundamental geographic information dataset of the National Geomatics Center of China (GS(2020)2075).

## 3 Results

### 3.1 Model accuracy

All 12 models ran successfully but showed clear differences in performance (**Figure 1**). Among them, RF, GBM, and GLM achieved the highest accuracy (Kappa > 0.8, ROC > 0.95, TSS > 0.85), indicating strong stability and predictive power, while ANN and SRE performed poorly. The ensemble model, which integrates superior algorithms, yielded a Kappa of 0.71, an ROC of 0.98, and a TSS of 0.88, all exceeding accepted accuracy thresholds. These results demonstrate that the integrated approach effectively reduced model uncertainty and provided reliable predictions of the potential distribution of *M. sativa* on the Qinghai–Tibet Plateau.

**Figure 1:**
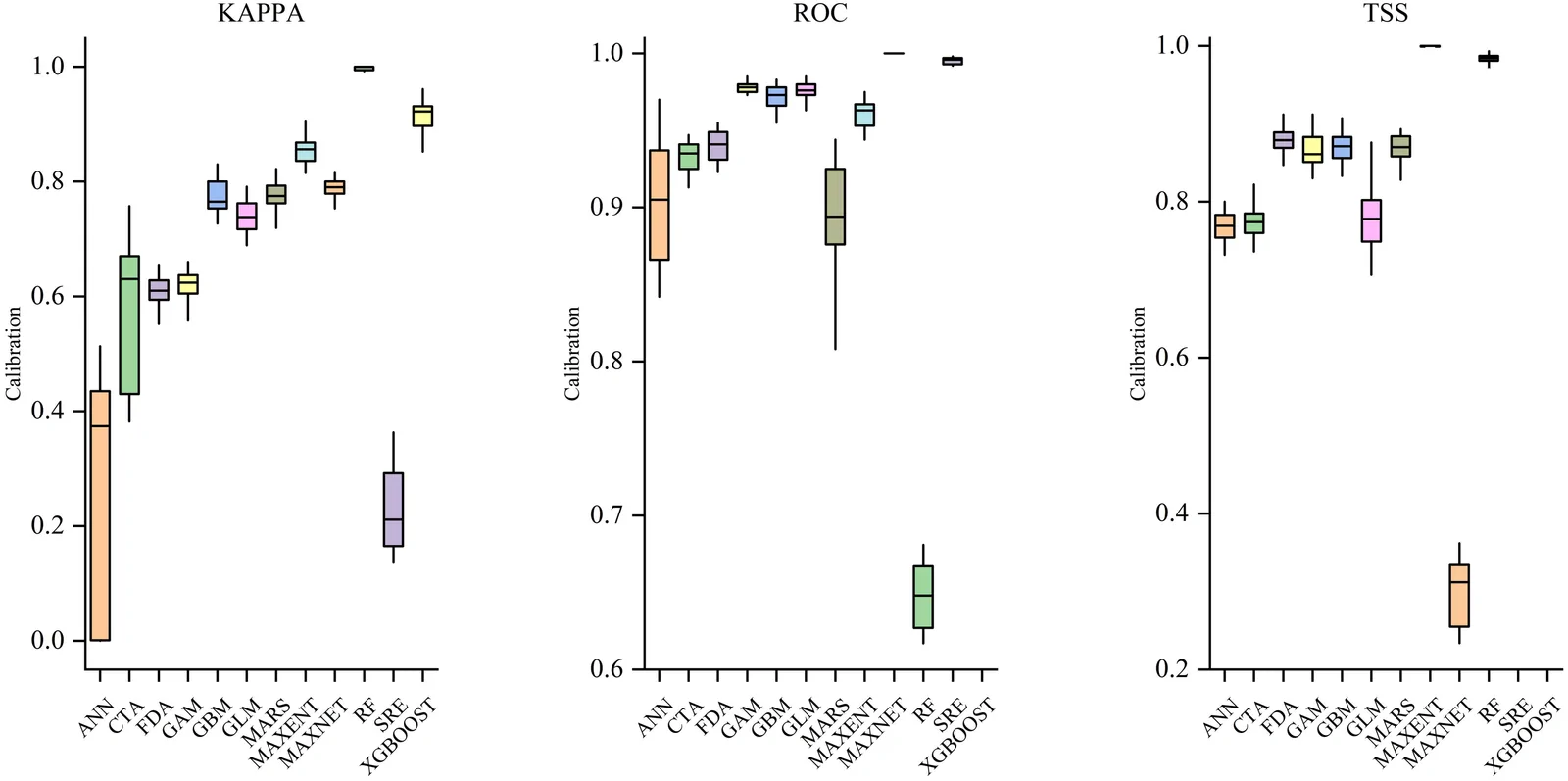
Evaluation scores of the individual models used in the ensemble modeling.

### 3.2 Environmental factors affecting the potential geographic distribution of *M. sativa*

The ensemble model revealed substantial differences in the contributions of environmental variables to the potential distribution of *M. sativa* (**Supplementary Figure 3**). Among the 18 predictors, elevation and temperature annual range (Bio7) contributed most, accounting for 20.5% and 18.4%, respectively, whereas mean diurnal range (Bio2) and mean temperature of the wettest quarter (Bio8) had relatively smaller effects. These results indicate that topographic and thermal factors are the dominant controls on the climatic suitability of M. sativa on the Qinghai-Tibet Plateau.

Response curves further revealed the species’ ecological preferences along these dominant gradients (**Figure 2**). The occurrence probability of *M. sativa* decreased markedly with increasing elevation, remaining above 0.65 below approximately 3,346.7 m and declining rapidly at higher altitudes. This pattern reflects the species’ preference for low- to mid-elevation environments, where temperatures and growing seasons are more favorable. In contrast, *M. sativa* exhibited a unimodal response to Bio7, with optimal suitability between 24.5 °C and 34.8 °C, beyond which its occurrence probability declined sharply. The occurrence probability showed a threshold response to Bio2 (mean diurnal range), remaining low below 14.1 °C and increasing sharply above this value, suggesting greater habitat suitability under larger diurnal temperature ranges. Similarly, occurrence probability increased with Bio8 (mean temperature of the wettest quarter), indicating that warmer conditions during the wettest quarter favor habitat suitability. Overall, the model results indicate that *M. sativa* prefers areas characterized by moderate elevation and stable, yet relatively broad, temperature amplitudes. These conditions typically correspond to the eastern and southern margins of the Plateau, where thermal resources are sufficient and topographic constraints are less pronounced.

**Figure 2:**
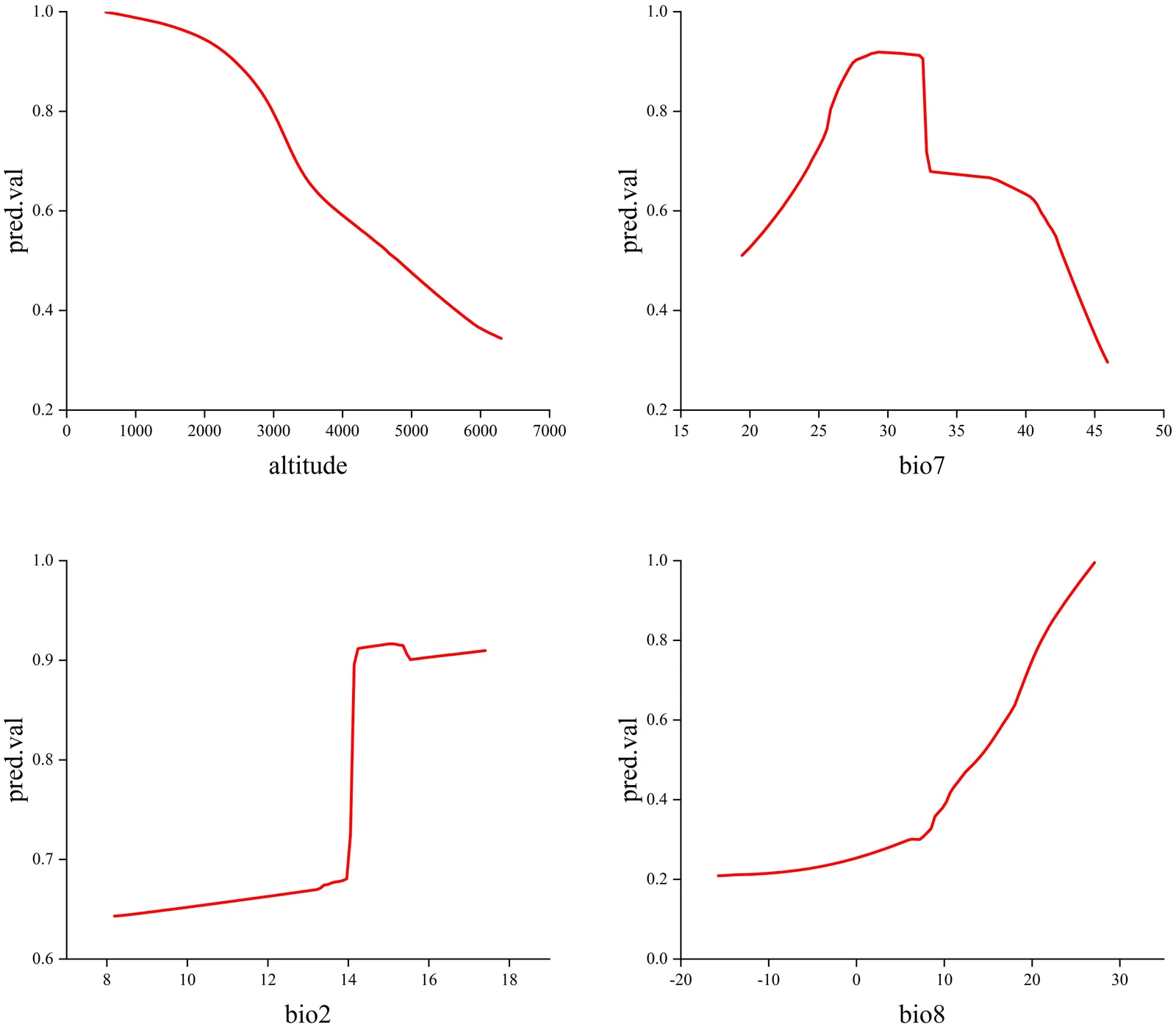
Response curves of *M. sativa* occurrence probability to key environmental variables. The y-axis represents the predicted occurrence probability of *M. sativa*.

### 3.3 Potential geographical distribution of *M. sativa* under current climatic conditions

The ensemble model predicted that under current climatic conditions, suitable habitats for *M. sativa* are primarily concentrated in the eastern and southern parts of the Qinghai–Tibet Plateau (**Figure 3**). High-suitability areas (11.77 × 10^4^ km^2^, **Table 1**) are distributed mainly across southeastern Tibet, southern Qinghai, and western Sichuan, extending along the Hengduan Mountains and the river valleys of the Yarlung Tsangpo and Jinsha Rivers.

**Figure 3:**
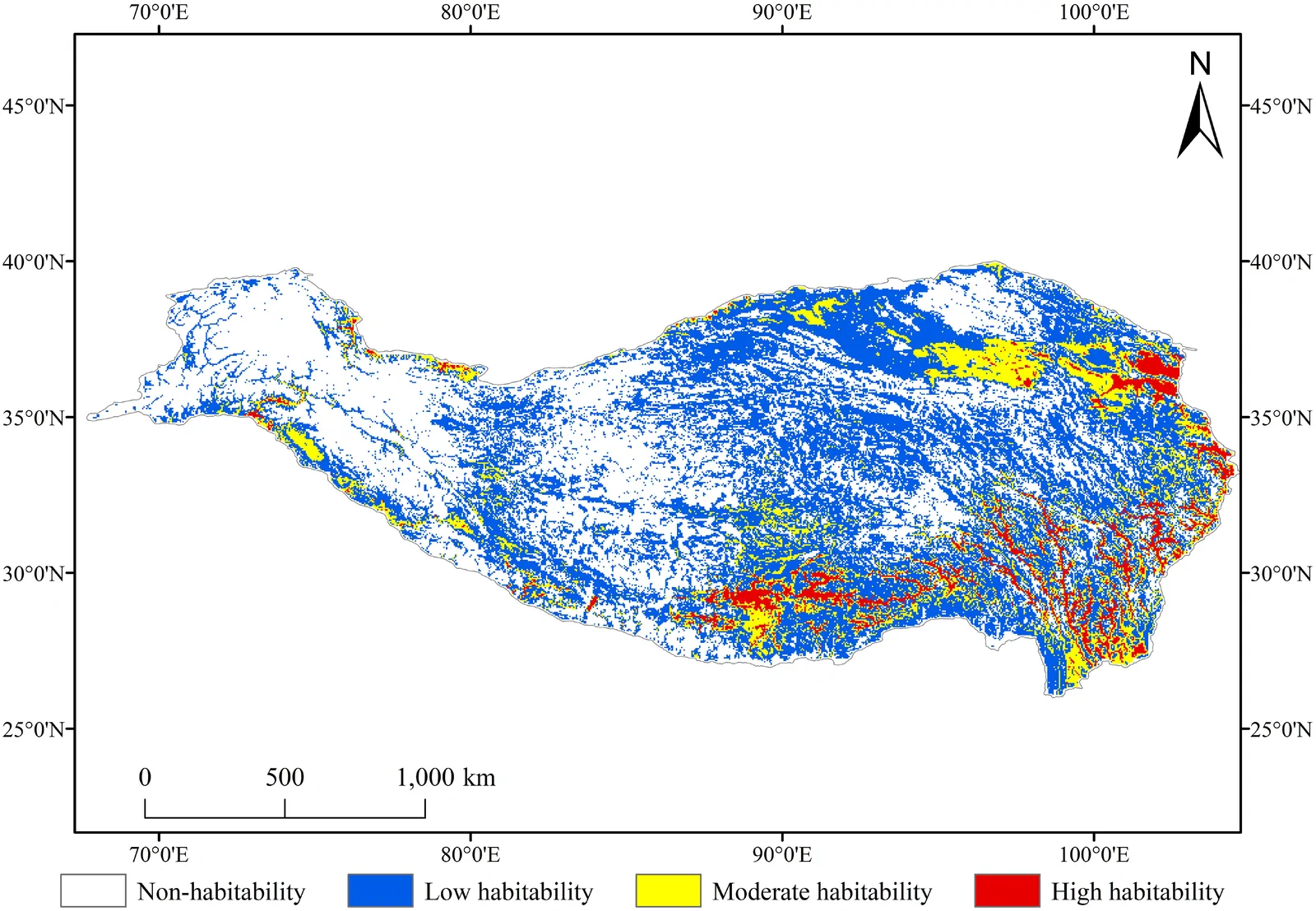
Potential geographical distribution of *M. sativa* under modern climate conditions.

**Table 1:** Area classification of suitable habitats for *M. sativa* under current and future climate scenarios for the 2050s and 2090s (10^4^km^2^).

Period	Low habitability area	Moderate habitability area	High habitability area	Total suitable area
Current	72.11	44.29	30.7	147.1
2050s-SSP126	85.81	58.04	37.86	181.71
2050s-SSP370	87.47	61.2	36.29	184.96
2050s-SSP585	76.22	59.27	36.87	172.36
2090s-SSP126	80.9	59.85	37.96	178.71
2090s-SSP370	97.95	69.56	36.95	204.46
2090s-SSP585	103.1	72.95	39.41	215.46

Moderate-suitability zones (29.48 × 10^4^ km^2^, **Table 1**) form transitional belts surrounding high-suitability areas, occurring in eastern Tibet, central and eastern Qinghai, and parts of southern Gansu. In contrast, low-suitability areas dominate the northern and western Plateau, where extreme elevation and cold, arid climates limit the establishment of *M. sativa*. Overall, the current distribution pattern aligns well with the known thermal map of the Chinese Plant Intelligence (https://www.iplant.cn), which supports the reliability of the ensemble simulation.

### 3.4 Changes in potential distribution patterns under different climate scenarios

The ensemble model projected substantial spatial adjustments in the suitable habitats of M. sativa on the Qinghai–Tibet Plateau under future climate scenarios (**Figure 4**, **Table 1**). Overall, the total suitable area increased markedly in both the 2050s and 2090s. In the 2050s, total suitable area reached 181.71 × 10^4^, 184.96 × 10^4^, and 172.36 × 10^4^ km² under SSP1-2.6, SSP3-7.0, and SSP5-8.5, respectively, representing increases of 17%–26% relative to the current 147.1 × 10^4^ km². Low-suitability areas increased to 85.81 × 10^4^, 87.47 × 10^4^, and 76.22 × 10^4^ km², while medium-suitability areas reached 58.04 × 10^4^, 61.20 × 10^4^, and 59.27 × 10^4^ km². High-suitability areas also expanded, but more modestly, to 37.86 × 10^4^, 36.29 × 10^4^, and 36.87 × 10^4^ km², respectively. Among the three scenarios, SSP3-7.0 produced the largest total suitable area in the 2050s, whereas SSP5-8.5 showed a slightly smaller expansion.

**Figure 4:**
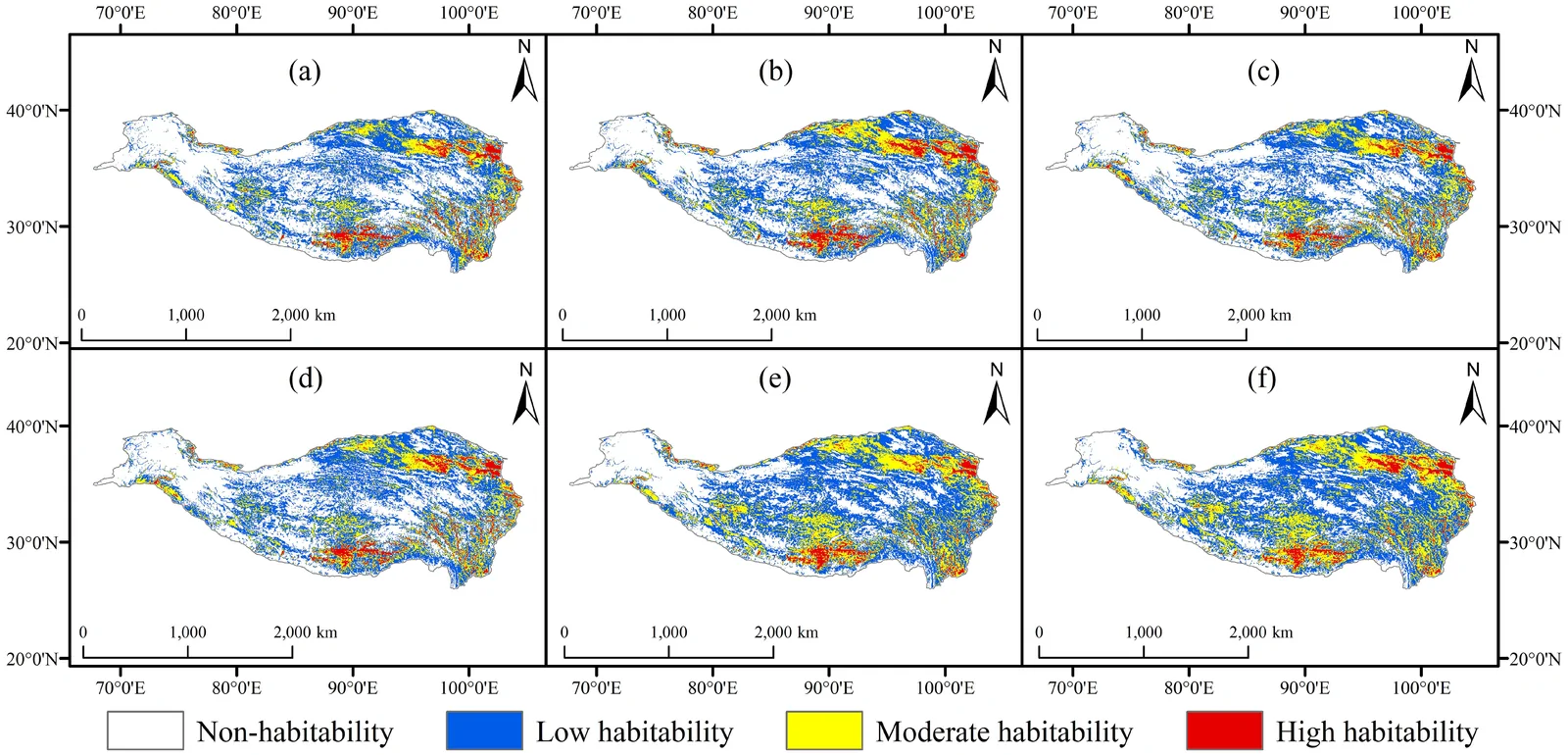
The color gradient indicates relative changes in habitat area, with cooler tones indicating expansion and warmer tones indicating reduction. **(a)** 2050s–SSP1-2.6, **(b)** 2050s–SSP3-7.0, **(c)** 2050s–SSP5-8.5, **(d)** 2090s–SSP1-2.6, **(e)** 2090s–SSP3-7.0, **(f)** 2090s–SSP5-8.5.

By the 2090s, differences among scenarios became more pronounced. Under SSP1-2.6, the total suitable area was 178.71 × 10^4^ km², remaining broadly stable relative to the 2050s. In contrast, it continued to increase under SSP3-7.0 and SSP5-8.5, reaching 204.46 × 10^4^ and 215.46 × 10^4^ km², respectively, equivalent to increases of 39% and 46.5% over the present. Under these two scenarios, low-suitability areas increased to 97.95 × 10^4^ and 103.1 × 10^4^ km², medium-suitability areas to 69.56 × 10^4^ and 72.95 × 10^4^ km², and high-suitability areas to 36.95 × 10^4^ and 39.41 × 10^4^ km², respectively.

Overall, future expansion was dominated by low- and medium-suitability habitats, whereas highly suitable habitat increased only from the current 30.7 × 10^4^ km² to a maximum of 39.41 × 10^4^ km². Accordingly, its share of total suitable habitat declined from 20.9% to approximately 18.3%, indicating that gains in high-quality habitat lag behind the broader expansion of climatically suitable areas.

Spatially, high- and moderate-suitability habitats expanded mainly along the northern and western margins of the Plateau, while low-suitability areas remained concentrated in the western and central regions (**Figure 4**). The overall increase in suitability was primarily driven by the conversion of low-suitability habitats to moderate-suitability habitats under intensified warming. Stable habitats dominated across all scenarios, accounting for more than 80% of the total suitable area, whereas habitat loss was mainly confined to southeastern Tibet and parts of southern Qinghai (**Figure 5**, **Table 2**). The greatest expansion occurred under SSP5-8.5 in the 2090s, with newly gained habitats reaching 49.91 × 10^4^ km² and habitat loss decreasing to 18.53 × 10^4^ km².

**Figure 5:**
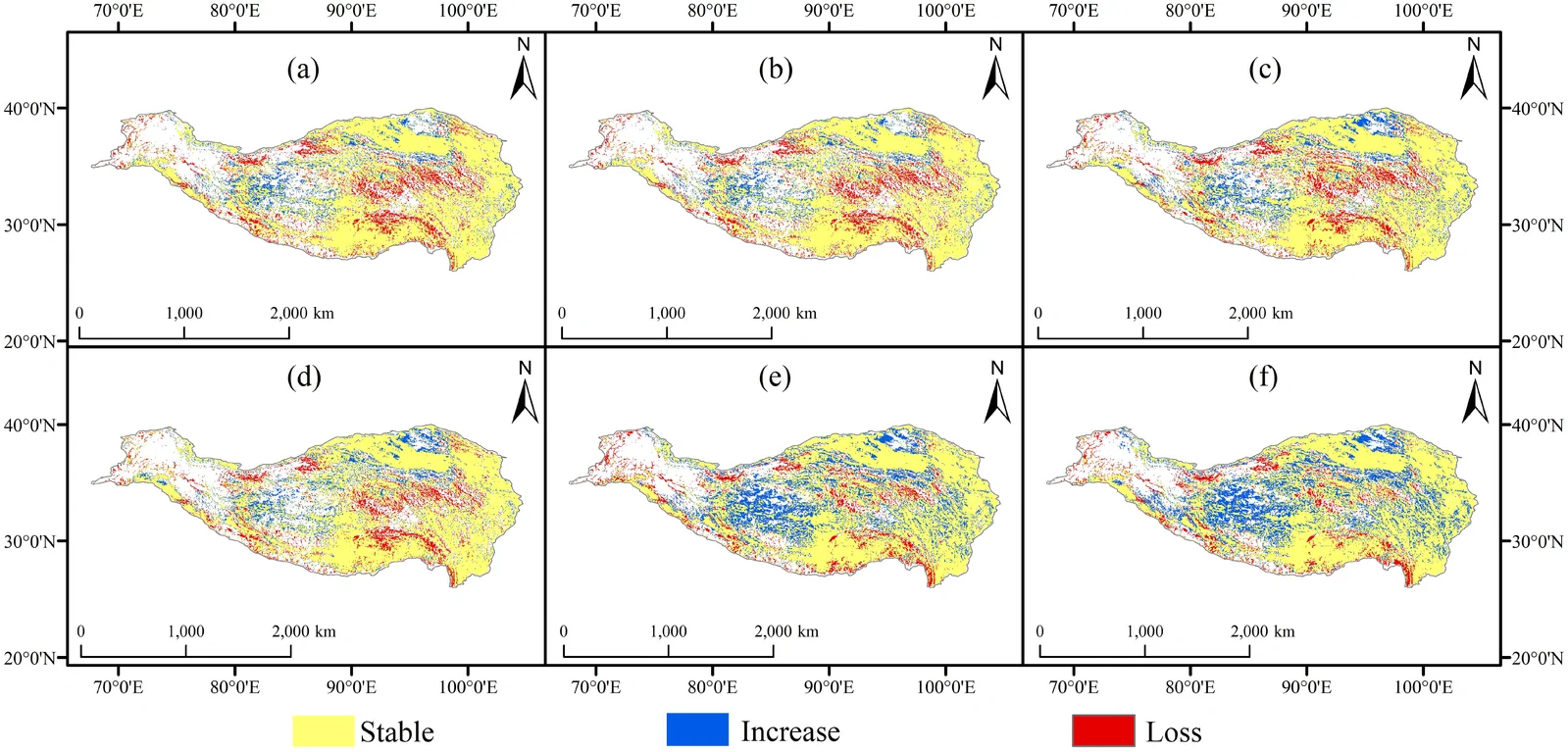
The color gradient represents the relative magnitude of change: warmer tones indicate habitat loss, yellow denotes stability, and cooler tones indicate expansion. **(a)** 2050s–SSP1-2.6, **(b)** 2050s–SSP3-7.0, **(c)** 2050s–SSP5-8.5, **(d)** 2090s–SSP1-2.6, **(e)** 2090s–SSP3-7.0, **(f)** 2090s–SSP5-8.5.

**Table 2:** Classification of habitat change for *M. sativa* under future climate scenarios for the 2050s and 2090s (10^4^ km^2^).

Period	Contraction	Stable	Expansion
2050s-SSP126	28.1	137.0	18.4
2050s-SSP370	27.0	138.1	26.2
2050s-SSP585	27.8	137.3	25.4
2090s-SSP126	25.8	139.3	21.2
2090s-SSP370	21.2	143.8	44.0
2090s-SSP585	18.5	146.6	49.9

### 3.5 Centroid migration of potential habitats

Centroid analysis of suitable habitats revealed a consistent northwestward shift across all scenarios (**Figure 6**). The current suitability centroid is located in southeastern Tibet and progressively migrates toward the interior of the Plateau under future warming. The magnitude of this displacement scales with emission intensity, being smallest under SSP1-2.6 and greatest under SSP5-8.5 by the 2090s, indicating that higher emissions accelerate the spatial displacement of climatically optimal conditions. Collectively, these projections suggest that *M. sativa* will retain a stable core habitat on the southeastern Plateau while progressively expanding into adjacent alpine areas under future climate scenarios.

**Figure 6:**
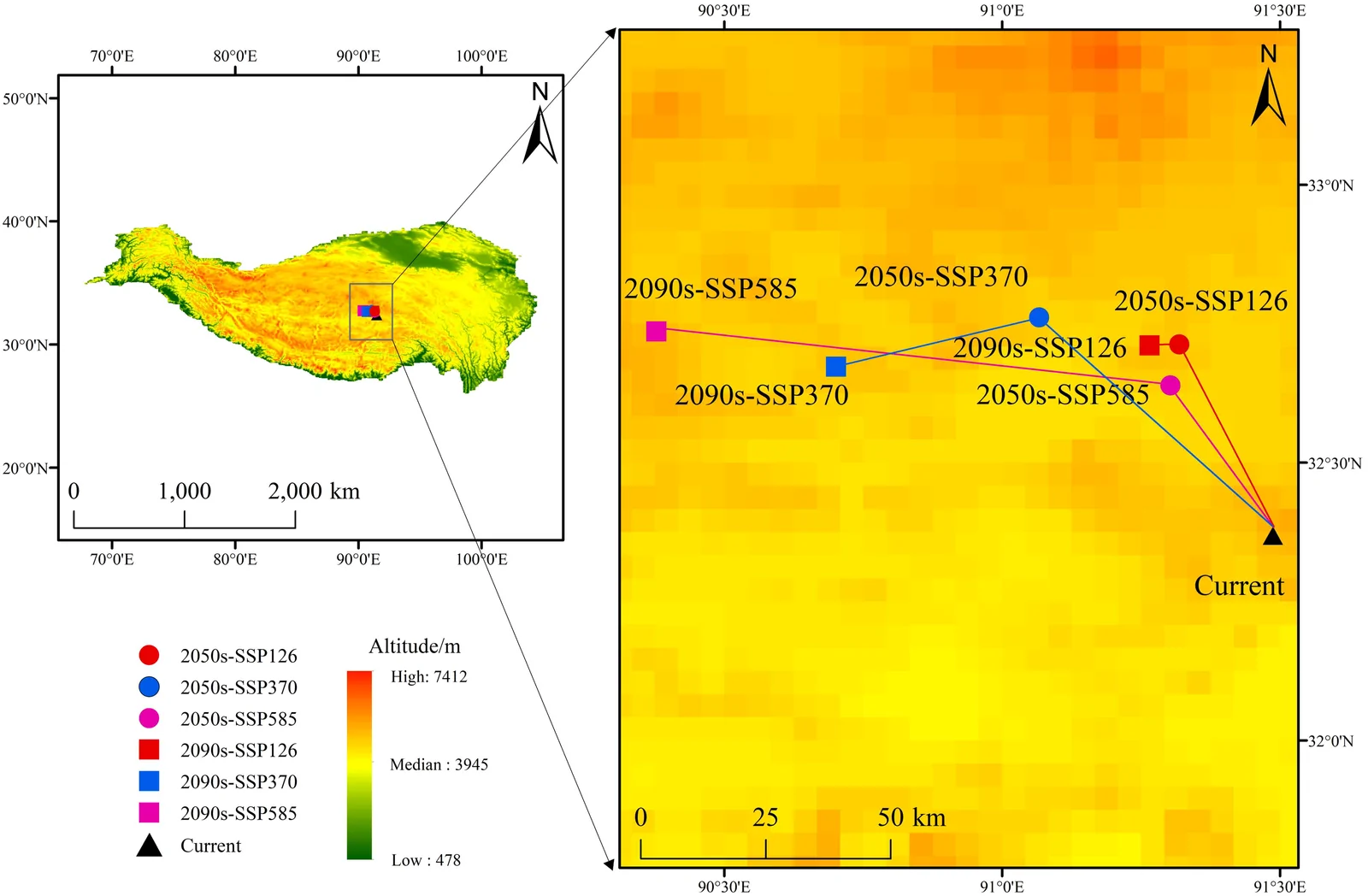
Changes in the gravity center of potential suitable habitat areas and the movement tendency of *M. sativa* under future climate change scenarios. The specific latitude and longitude coordinates of the centroids under each scenario are as follows: current (91.3°E, 32.2°N), 2050s SSP1-2.6 (91.5°E, 32.3°N), 2050s SSP3-7.0 (92.1°E, 32.7°N), 2050s SSP5-8.5 (91.2°E, 33.4°N), 2090s SSP1-2.6 (91.2°E, 32.5°N), 2090s SSP3-7.0 (90.4°E, 33.5°N), 2090s SSP5-8.5 (90.2°E, 32.8°N).

## 4 Discussion

### 4.1 The impact of environmental and species data on model accuracy

The performance of Species Distribution Models (SDMs) is strongly influenced by input-data quality and model structure (Friedman et al., 2000; Searcy and Shaffer, 2016; Xie et al., 2023). Although spatial uncertainty was not explicitly quantified using the coefficient of variation across algorithms, ensemble averaging can reduce model-specific uncertainty by weighting individual models according to predictive performance (Thuiller et al., 2009). The ensemble model constructed from 92 valid occurrence records of M. sativa achieved high ROC, TSS, and Kappa values (**Figure 1**), indicating robust predictive performance. This reliability likely reflects the adequate occurrence dataset, comprehensive environmental predictors, and integration of multiple algorithms to reduce individual-model bias (Pecchi et al., 2019). However, these projections represent climatic suitability rather than realized distribution or remediation efficacy. Although *M. sativa* possesses traits favorable for heavy-metal stabilization, including high biomass, deep rooting, and metal-binding capacity, its actual remediation performance also depends on soil contamination levels, metal bioavailability, and field management, which were not empirically assessed here. Therefore, this study provides baseline climatic-suitability information as a prerequisite for future evaluation of the species’ remediation potential in heavy-metal-contaminated areas of the Qinghai–Tibet Plateau.

The density of species distribution data has a significant impact on model stability. Limited sample sizes can increase sensitivity to outliers and restrict representation of the full climatic niche. Although the 92 occurrence records used here meet the basic requirements for ensemble SDM applications, this sample size remains relatively small for a large-scale analysis, particularly in regions where *M. sativa* is rare or under-sampled (Pecchi et al., 2019; Naimi et al., 2014; Xie et al., 2023). Nevertheless, the high predictive performance (ROC = 0.98, TSS = 0.88) and ensemble framework partially mitigate this limitation by reducing model-specific bias. Careful control of model complexity and screening of correlated predictors further improved model stability across environmental gradients.

Another important limitation is that the occurrence records may partly reflect cultivation history rather than purely climatic suitability, as *M. sativa* is widely cultivated as a forage crop. This anthropogenic influence may bias the estimated climatic niche and should be considered when interpreting projected suitability in natural habitats (Liu, 2025). In addition, model evaluation relied solely on cross-validation without an independent external dataset. Residual spatial autocorrelation among occurrence records may therefore have inflated performance metrics, and this was not explicitly assessed using statistics such as Moran’s I. Future studies should incorporate spatially independent validation datasets and autocorrelation-correction approaches to improve the robustness and transferability of projections.

Overall, combining screened occurrence data with carefully selected environmental predictors improved model reliability, but the above uncertainties should be considered when interpreting the results. Reliable SDM projections nevertheless provide an essential ecological basis for phytoremediation planning, because *M. sativa* can only be considered for Cd remediation where climatic conditions permit its persistent establishment and population stability on the Qinghai–Tibet Plateau.

### 4.2 The influence of environmental variables on the potential geographical distribution of *M. sativa*

The ensemble model identified elevation and annual temperature range (Bio7) as the primary constraints on the potential distribution of *M. sativa* across the Qinghai–Tibet Plateau (**Supplementary Figure 3**). This pattern is consistent with the Plateau’s extreme topoclimatic conditions, characterized by low mean temperatures, large temperature fluctuations, and intense solar radiation, which impose substantial thermal stress on vegetation (Guo et al., 2020; Shen et al., 2022). In such environments, elevation strongly regulates temperature and moisture availability and thus plays a key role in shaping the climatic niche of *M. sativa*.

Although *M. sativa* is broadly adaptable and widely distributed across continents, its occurrence in alpine regions remains strongly constrained by elevation. Chen et al. (2023) demonstrated its tolerance and soil-improvement potential at high altitudes, whereas Lei (2021) reported significant yield declines with increasing elevation. Records from the National Plant Digital Specimen Resource (http://www.cvh.ac.cn/) further indicate that *M. sativa* occurrences on the Qinghai–Tibet Plateau are concentrated in the relatively low-elevation eastern and southern regions, supporting the topographic constraint identified by our model. The dominant influence of elevation likely reflects its integrated effects on temperature and water availability. With every 100 m increase in elevation, air temperature decreases by approximately 0.6 °C, accompanied by a greater diurnal temperature range and reduced effective accumulated temperature, which together constrain photosynthetic performance and biomass accumulation in *M. sativa*. The species grows optimally at 15–25 °C, while growth is strongly inhibited when nighttime temperatures approach 5 °C. Consequently, areas above approximately 3,300 m become progressively less suitable because of low-temperature stress. The importance of Bio7 (annual temperature range) further indicates the sensitivity of *M. sativa* to temperature seasonality. As a species originating from temperate regions, *M. sativa* has developed phenological adaptations to seasonal temperature fluctuations; however, an excessively large annual temperature range (>45 °C) implies exposure to both severe winter cold and extreme summer heat, potentially causing physiological damage and shortening the effective growing season (Liu, 2025).

The optimal Bio7 range identified in this study is consistent with the temperate climatic niche of *M. sativa*, suggesting that excessive annual temperature variability may hinder completion of its normal life cycle. This interpretation aligns with experimental observations showing that *M. sativa* tolerates arid, thermally variable environments (Duan et al., 2024) and achieves optimal germination within specific temperature amplitudes (Zhu et al., 2020).To our knowledge, this study is the first to evaluate the climatic suitability of *M. sativa* across the entire Qinghai–Tibet Plateau and to identify elevation and temperature seasonality as its dominant environmental constraints. Overall, the predominance of topographic and thermal variables suggests that the distribution of *M. sativa* on the Plateau is largely governed by elevation-associated thermal conditions. From a phytoremediation perspective, these climatic constraints have direct practical implications because remediation efforts are unlikely to be sustainable where *M. sativa* cannot establish persistent populations. Although our results represent potential climatic suitability rather than realized distribution, they provide an important basis for assessing the ecological applicability of *M. sativa* and for guiding its use in soil restoration and heavy-metal remediation in alpine regions.

### 4.3 Changes in the potential geographic distribution of *M. sativa* in the context of climate change

The ensemble projections indicate moderate but spatially distinct shifts in the potential distribution of *M. sativa* on the Qinghai–Tibet Plateau. Although the total suitable area is projected to remain relatively stable, highly and moderately suitable habitats are expected to expand under future emission scenarios, largely through the conversion of marginally suitable areas. Meanwhile, the suitability centroid is projected to shift northwestward and toward higher elevations, consistent with the poleward and upslope redistribution of species commonly observed under climate warming (Hao et al., 2024; Mao et al., 2024; Thuiller et al., 2005).

These shifts are likely driven primarily by changes in thermal conditions, particularly temperature annual range (Bio7), a key determinant of the climatic suitability of *M. sativa*. Under the SSP5-8.5 scenario, substantial warming is projected by the late 21st century (Lee et al., 2023), which may extend frost-free periods and growing seasons in alpine regions. Such warming could facilitate the establishment of *M. sativa* in newly suitable high-elevation areas. Similar distributional shifts have been reported for H. perforatum (Hao et al., 2024) and Achillea spp (Mao et al., 2024), suggesting that ongoing warming may promote the expansion of cold-tolerant species into previously unsuitable alpine habitats.

The projected expansion of climatically suitable areas under future warming suggests that *M. sativa* may retain or even increase its potential for Cd remediation on the Qinghai–Tibet Plateau. As climatic constraints weaken at higher elevations, newly suitable areas may support persistent populations and expand the spatial scope of phytoremediation. This potential is reinforced by functional traits favorable for soil rehabilitation, including high biomass, deep rooting, and strong metal-binding capacity, which promote the stabilization of contaminated soils. Thus, warming may enhance the phytostabilization potential of *M. sativa* in regions where land degradation and mining-related pollution overlap.

However, this apparent advantage contrasts with the increasing extinction risk projected for many alpine taxa (Thomas et al., 2015) and should be interpreted cautiously. Our projections consider climatic suitability only and do not account for biotic interactions with native alpine vegetation. Moreover, introducing *M. sativa* into previously unoccupied alpine habitats may increase invasion risk and threaten local plant diversity (Fayaz et al., 2026). Therefore, its use in ecological restoration should be accompanied by ecological risk assessment and consideration of local conservation priorities.

Nevertheless, these projections reflect climatic suitability rather than realized distribution. Non-climatic factors, including land-use change, grazing pressure, and local soil contamination, may further constrain actual establishment. Future assessments should therefore incorporate dynamic soil and anthropogenic variables to improve predictions and better characterize the interactions between climate adaptation and remediation performance in *M. sativa*.

### 4.4 Implications for ecological restoration and management

The projected persistence and northward expansion of *M. sativa* under future climate scenarios may enhance its value for ecological restoration on the Qinghai–Tibet Plateau. As warming relaxes thermal constraints at high elevations, previously marginal areas may become suitable for establishment. Many of these areas are affected by mining, grazing, and infrastructure development, increasing the need for vegetation recovery. The expansion of climatically suitable habitat may therefore broaden opportunities to use *M. sativa* in the rehabilitation of degraded alpine soils.

*M. sativa* also possesses traits favorable for phytostabilization and soil improvement, including a deep, fibrous root system, metal-immobilizing capacity, and biological nitrogen fixation. These traits can improve soil structure, promote nutrient cycling, and reduce heavy-metal mobility. However, restoration outcomes will also depend on local edaphic conditions, particularly soil metal concentrations, pH, and organic matter content. Effective management should therefore combine climate-based suitability projections with site-specific soil assessments and long-term monitoring of species performance and remediation outcomes under changing thermal and hydrological conditions.

Overall, *M. sativa* represents a potentially valuable multifunctional species for linking climate adaptation with pollution control. Its projected persistence and expansion under future warming support its potential inclusion in restoration strategies aimed at improving ecosystem resilience in degraded alpine environments.

### 4.5 Research Prospects

Despite the overall robustness of the ensemble projections, several sources of uncertainty should be acknowledged when interpreting the results. The analysis was based on 92 occurrence records, which provide adequate information for model calibration but remain limited relative to the spatial extent and environmental heterogeneity of the Qinghai–Tibet Plateau. In addition, some records may represent cultivated populations rather than natural occurrences, potentially affecting the characterization of the species’ realized environmental niche. Future projections were also derived from a single global climate model (BCC-CSM2-MR), and therefore do not incorporate uncertainty arising from differences among GCMs. Furthermore, although the models considered climatic, topographic, edaphic, and ultraviolet radiation variables, other factors relevant to establishment and remediation performance, including soil heavy-metal concentrations, land use, and grazing intensity, were not explicitly represented. Spatial thinning and cross-validation were applied to reduce overfitting, but residual spatial autocorrelation was not independently evaluated, and predictive performance was not tested against an external validation dataset. These uncertainties do not negate the projected distributional patterns, but they should be considered when translating climatic suitability into restoration and management decisions. Future work incorporating multi-GCM ensembles, independent validation, spatial autocorrelation assessment, and additional non-climatic variables would help improve the robustness and practical relevance of the projections.

## 5 Conclusions

By integrating multiple modeling algorithms, this study characterized the potential distribution of *M. sativa* on the Qinghai–Tibet Plateau and its response to future climate change. Elevation and temperature annual range (Bio7) emerged as the dominant environmental determinants of climatic suitability. Under current conditions, suitable habitats are concentrated mainly in the eastern and southern Plateau. In contrast, future warming is projected to maintain the overall suitable area while increasing highly suitable habitats toward the northwest and at higher elevations. These shifts indicate that *M. sativa* may retain substantial climatic suitability under future warming, particularly as thermal constraints weaken in alpine environments. Together with its high biomass and capacity for metal accumulation and stabilization, this climatic adaptability highlights the potential of *M. sativa* for phytoremediation and ecological restoration of degraded alpine soils. However, the projections describe potential suitability rather than realized distribution and remain subject to uncertainties associated with cultivated occurrence records, the use of a single GCM, and unmodeled local factors such as soil metal concentrations, pH, land use, and biotic interactions. Future assessments integrating multi-GCM ensembles, soil contamination data, species interactions, and field-based monitoring will be essential for refining restoration suitability and improving the reliability of phytoremediation planning on the Qinghai–Tibet Plateau.

## Data Availability

The original contributions presented in the study are included in the article/**Supplementary Material**. Further inquiries can be directed to the corresponding authors.
