# Supplementary material for: Climatic suitability and distribution prediction of Medicago sativa on the Qinghai–Tibet Plateau: an ensemble species distribution modeling approach
Source: Front Plant Sci. 2026 Sep 14;17:1930959. doi: 10.3389/fpls.2026.1930959 (PMC13617093; doi:10.3389/fpls.2026.1930959)
Supplement: Supplementary file 1 [file DataSheet1.docx]

Supplementary Material

## 1.1 Supplementary Figures

Supplementary Figure S1 Morphological characteristics of *M. sativa*

Supplementary Figure S2 The geographical distribution of *M. sativa.*

Supplementary Figure S3.Contribution rate of environmental variable on the distribution of *M. sativa*

## 1.2 Supplementary Tables

Supplementary Table S1 Environmental variables used in the research institute

| type | factor | description | unit |
| --- | --- | --- | --- |
| Bioclimatic variable | bio1 | Annual Mean Temperature* | ℃ |
| Bioclimatic variable | bio2 | Mean Diurnal Range* | ℃ |
| Bioclimatic variable | bio3 | Isothermality* | - |
| Bioclimatic variable | bio4 | Temperature Seasonality | - |
| Bioclimatic variable | bio5 | Max Temperature of Warmest Month | ℃ |
| Bioclimatic variable | bio6 | Min Temperature of Coldest Month | ℃ |
| Bioclimatic variable | bio7 | Temperature Annual Range* | ℃ |
| Bioclimatic variable | bio8 | Mean Temperature of Wettest Quarter* | ℃ |
| Bioclimatic variable | bio9 | Mean Temperature of Driest Quarter* | ℃ |
| Bioclimatic variable | bio10 | Mean Temperature of Warmest Quarter | ℃ |
| Bioclimatic variable | bio11 | Mean Temperature of Coldest Quarter | ℃ |
| Bioclimatic variable | bio12 | Annual Precipitation | mm |
| Bioclimatic variable | bio13 | Precipitation of Wettest Month | mm |
| Bioclimatic variable | bio14 | Precipitation of Driest Month* | mm |
| Bioclimatic variable | bio15 | Precipitation Seasonality* | mm |
| Bioclimatic variable | bio16 | Precipitation of Wettest Quarter | mm |
| Bioclimatic variable | bio17 | Precipitation of Driest Quarter | mm |
| Bioclimatic variable | bio18 | Precipitation of Warmest Quarter* | mm |
| Bioclimatic variable | bio19 | Precipitation of Coldest Quarter* | mm |
| Topographic variable | alt | altitude* | m |
| Topographic variable | asp | aspect* | - |
| Topographic variable | slo | slope* | ° |
| Soil variable | awc_class | available water content class* | - |
| Soil variable | t_oc | topsoil organic carbon* | mg/L |
| Soil variable | t_ph | topsoil pH* | - |
| Soil variable | s_oc | subsoil organic carbon* | mg/L |
| Soil variable | s_ph | subsoil pH* | - |
| UV radiation intensity | uv-b | ultraviolet radiation b* | nm |

Note: * is the modeling variable

Supplementary Table S2 Geographic distribution records of *M. sativa* before filtering

| No. | Longitude | Latitude | No. | Longitude | Latitude | No. | Longitude | Latitude |
| --- | --- | --- | --- | --- | --- | --- | --- | --- |
| 1 | 96.92436 | 30.05903 | 46 | 102.80749 | 35.88202 | 91 | 92.2089 | 29.0914 |
| 2 | 98.59968 | 29.68578 | 47 | 102.71337 | 36.09046 | 92 | 90.7525 | 29.3092 |
| 3 | 91.69010 | 29.03087 | 48 | 101.79554 | 36.46007 | 93 | 89.8497 | 29.2192 |
| 4 | 92.46896 | 28.41402 | 49 | 102.75010 | 35.84472 | 94 | 88.9261 | 29.1942 |
| 5 | 79.80932 | 31.48534 | 50 | 101.43953 | 36.04616 | 95 | 88.9606 | 29.1783 |
| 6 | 94.21994 | 29.22092 | 51 | 101.70827 | 36.64488 | 96 | 88.8395 | 29.3246 |
| 7 | 95.77390 | 29.86506 | 52 | 97.89430 | 35.99969 | 97 | 88.7672 | 29.1916 |
| 8 | 101.26245 | 36.68859 | 53 | 99.30735 | 28.24485 | 98 | 88.6656 | 29.1464 |
| 9 | 102.83639 | 36.32556 | 54 | 98.85222 | 28.42422 | 99 | 89.1644 | 29.3919 |
| 10 | 102.40857 | 36.48782 | 55 | 99.31007 | 28.24577 | 100 | 89.8950 | 29.2306 |
| 11 | 102.27045 | 36.10099 | 56 | 98.92446 | 28.47009 | 101 | 88.4476 | 29.1763 |
| 12 | 102.49538 | 35.85667 | 57 | 98.91864 | 28.49143 | 102 | 87.5761 | 29.0989 |
| 13 | 100.25962 | 38.18292 | 58 | 98.91232 | 28.47892 | 103 | 92.0227 | 29.2587 |
| 14 | 102.04699 | 35.94913 | 59 | 98.92458 | 28.46990 | 104 | 92.0351 | 29.2630 |
| 15 | 100.62662 | 36.28870 | 60 | 100.07947 | 27.05219 | 105 | 92.0636 | 29.2887 |
| 16 | 101.43953 | 36.04616 | 61 | 102.88252 | 35.72350 | 106 | 91.4522 | 29.2581 |
| 17 | 99.11673 | 30.01166 | 62 | 98.581803 | 39.669881 | 107 | 91.2672 | 29.2650 |
| 18 | 103.17351 | 31.44255 | 63 | 100.20640 | 30.95491 | 108 | 100.810000 | 38.460000 |
| 19 | 103.85959 | 31.68681 | 64 | 100.20552 | 30.98916 | 109 | 80.05 | 35.3 |
| 20 | 103.61058 | 32.66180 | 65 | 78.21225 | 39.50073 | 110 | 79.133333 | 36.6 |
| 21 | 102.07061 | 31.48201 | 66 | 74.478764 | 39.787400 | 111 | 82.366667 | 35.333333 |
| 22 | 102.36942 | 31.00221 | 67 | 77.296667 | 40.261017 | 112 | 79.833333 | 36.983333 |
| 23 | 102.99652 | 32.06781 | 68 | 79.224083 | 41.185064 | 113 | 81.430000 | 36.52000 |
| 24 | 102.21350 | 31.91175 | 69 | 101.48111 | 36.49305 | 114 | 81.320000 | 36.510000 |
| 25 | 102.97439 | 33.58438 | 70 | 95.827853 | 31.273609 | 115 | 82.420000 | 37.050000 |
| 26 | 101.96356 | 30.00441 | 71 | 85.295858 | 28.8477 | 116 | 79.450000 | 37.020000 |
| 27 | 102.24143 | 29.92040 | 72 | 103.874624 | 34.219102 | 117 | 80.470000 | 37.020000 |
| 28 | 101.51342 | 29.00643 | 73 | 82.250000 | 32.250000 | 118 | 98.792056 | 28.569194 |
| 29 | 101.13154 | 30.98533 | 74 | 91.0894 | 29.2789 | 119 | 99.304972 | 28.245306 |
| 30 | 100.68240 | 31.39799 | 75 | 89.5849 | 28.9045 | 120 | 98.88346 | 28.44642 |
| 31 | 99.80506 | 28.93673 | 76 | 89.5283 | 28.9694 | 121 | 93.416667 | 29.833333 |
| 32 | 99.29299 | 28.71878 | 77 | 89.8146 | 28.8485 | 122 | 88.850000 | 29.266667 |
| 33 | 99.72456 | 27.82609 | 78 | 89.6739 | 28.5811 | 123 | 101.866667 | 36.533333 |
| 34 | 98.92446 | 28.47009 | 79 | 89.3994 | 29.0542 | 124 | 98.68542 | 28.76808 |
| 35 | 102.88252 | 35.72350 | 80 | 87.5214 | 29.1658 | 125 | 98.69168 | 28.72692 |
| 36 | 104.37749 | 33.79081 | 81 | 87.6122 | 29.1014 | 126 | 98.69132 | 28.72726 |
| 37 | 88.17532 | 39.02899 | 82 | 91.7400 | 29.1656 | 127 | 99.31007 | 28.24577 |
| 38 | 75.26534 | 39.72512 | 83 | 90.2381 | 29.4225 | 128 | 99.35000 | 37.05000 |
| 39 | 77.42010 | 37.88917 | 84 | 90.1728 | 29.4422 | 129 | 98 | 33.716667 |
| 40 | 79.92851 | 37.12045 | 85 | 88.3753 | 29.2114 | 130 | 99.810000 | 27.530000 |
| 41 | 80.81320 | 37.00412 | 86 | 89.1617 | 29.3919 | 131 | 99.290000 | 36.660000 |
| 42 | 82.70271 | 37.07026 | 87 | 89.1640 | 29.3919 | 132 | 91.100000 | 29.600000 |
| 43 | 94.071667 | 28.234258 | 88 | 91.7139 | 29.1389 | 133 | 101.770000 | 36.620000 |
| 44 | 91.322325 | 29.260122 | 89 | 91.7050 | 29.1044 | 134 | 102.580000 | 36.300000 |
| 45 | 91.736450 | 29.244600 | 90 | 92.2086 | 29.0714 | 135 | 89.460000 | 29.450000 |
